# Genotypic characterization of *mec*A gene and antibiogram profile of coagulase-negative staphylococci in subclinical mastitic cows

**DOI:** 10.14202/vetworld.2022.2186-2191

**Published:** 2022-09-15

**Authors:** Eman S. Ibrahim, Sohad M. Dorgham, Asmaa S. Mansour, Abeer M. Abdalhamed, Doaa D. Khalaf

**Affiliations:** 1 Department of Microbiology and Immunology, National Research Centre, Giza, Egypt; 2 Department of Parasitology and Animals Diseases, National Research Centre, Giza, Egypt

**Keywords:** coagulase-negative staphylococci, cows, *mecA* gene, polymerase chain reaction, subclinical mastitis

## Abstract

**Background and Aim:**

Coagulase-negative staphylococci (CNS) are becoming the major cause of clinical and subclinical bovine mastitis around the world. This study aims to estimate the prevalence, antibiogram, and frequency of the methicillin-resistant (MR) (*mecA*) gene in CNS collected from cows with subclinical mastitis

**Materials and Methods:**

Thirty-four milk samples were collected from 20 cows. Fifteen subclinical mastitis samples (~44.12%) were identified as CNS isolates. The Vitek2 compact system method was employed for the identification of the species. Furthermore, antibiotic sensitivity tests were performed against 10 different antibiotics for CNS strains. The *mecA* gene from isolated CNS was detected by conventional polymerase chain reaction (PCR).

**Results:**

*Staphylococcus haemolyticus* was the most predominant isolated species with an incidence of 33.3% (5/15 isolates), followed by 26.7% for *Staphylococcus sciuri* and *Staphylococcus vitamins* (4/15 isolates), and 13.3% for *Staphylococcus vitulinus* (2/15 isolates), respectively. The highest resistance rates were determined to be 40% (6/15 isolates) against penicillin and oxacillin (OX), 33.3% (5/15 isolates) against clindamycin, 13% (2/15 isolates) against chloramphenicol, amoxicillin, and erythromycin, and 5% (1/15 isolates) against ciprofloxacin, respectively. The results revealed that the isolates were resistant to one or more antimicrobial agents, with five isolates displaying multiple antimicrobial resistance. Furthermore, the results exhibit that all CNS isolates had the *mecA* gene at 310 bp with a 100% frequency. Moreover, for detecting MR isolates, there are significant discrepancies between phenotypic and genotypic approaches, and only 6/15 CNS isolates phenotypically demonstrated OX resistance.

**Conclusion:**

The results emphasize the necessity of frequent monitoring of phenotypic and genotypic profiles of CNS isolates to ensure effective control measures and the prevention of multidrug resistance strain evolution.

## Introduction

Mastitis remains one of the most common health problems in animals, causing significant financial losses to breeders. Mastitis can be caused by both infectious and non-infectious factors. Moreover, nearly 95% of cases are caused by microbial and pathogenic microorganisms that enter the teat canal through the mammary gland [1]. In recent years, coagulase-negative staphylococci (CNS) have attracted a lot of attention in both human and veterinary medicines. They have been linked to certain types of infections such as urinary tract, bloodstream, and invasive device-related infections [2]. Furthermore, CNS is a well-known cause of mastitis worldwide, where they were previously thought to be opportunistic pathogens that cause mild mastitis which is usually subclinical. Their importance in intramammary infections (IMIs) is growing, as CNS species have been isolated from the majority of IMIs [3]. The CNS caused a protracted intramammary infection, an increase in somatic cell counts, and a decrease in milk and other dairy product production and quality; therefore, they are now designated as emergent bovine mastitis pathogens causing a series of economic losses to the livestock industry [4]. These bacteria are regarded as reservoirs of resistance genes since they are now showing an increase in antimicrobial resistance [5]. Antimicrobial resistance is a major problem for a variety of reasons, including treatment failure, financial losses, and public health consequences. Furthermore, resistant pathogens have the potential to spread and become a major worry in health centers and communities [6].

Hence, it is critical to precisely identify the bacteria involved before choosing an antibiotic medication. In addition, it is critical to consider the susceptibility of the microorganism, the drug’s pharmacokinetics, the duration of treatment at the infection site, and toxicity in the animals when developing ways to prevent the development of antimicrobial resistance and decrease the risk of transmission. The β-lactams antibiotics are commonly utilized in intramammary infusion therapy [7]. Moreover, the methicillin-resistant (MR) staphylococci are resistant to all β-lactam antibiotics due to the acquisition of penicillin (P)-binding protein (PBP)2a, a PBP with poor affinity for these medicines. This low-affinity protein is encoded by the *mecA/C* genes located on a mobile genetic element called staphylococcal chromosomal cassette (SCCmec). The SCCmec has been classified into 13 different types (types I–XIII) [8]. The MR staphylococci pose a threat to both human and animal health as they demonstrate coresistance to additional beta-lactam antibiotics such as oxacillin (OX)/methicillin, as well as aminoglycosides, tetracyclines (TEs), macrolides, chloramphenicol (C), fluoroquinolones, and rifampicin [9]. The CNS is thought to have a greater proportion of methicillin resistance than *Staphylococcus aureus* [10]. The MR CNS species contain the *mecA* gene, which has been horizontally transferred among staphylococci (MR-CNS). Furthermore, mecA-positive CNS could be potential donors for the spread of new MR *S. aureus* (MRSA) clones [11].

This study aimed to analyze the antimicrobial resistance patterns of CNS isolated from milk samples from subclinical mastitic cows and the detection of MR isolates through the molecular typing technique.

## Materials and Methods

### Ethical approval

This study was approved by Ethical Committee for Medical Research (no. 12020232/2019) at the National Research Centre, Egypt.

### Study period and location

The study was conducted from May to August 2021 at National Research Center, Dokki, Egypt. The samples were examined at the National Research Center’s Microbiology and Immunology Department Laboratory, which is a part of the Veterinary Research Division.

### Sample collection

A total of 20 mixed breed dairy cows, ranging in age from 3 to 7 years old, were investigated in private dairy farms at Giza Governorate, Egypt. The farm failed to take the necessary hygienic precautions to prevent mastitis and other infectious illnesses. The milking procedure was carried out in the usual manner. The California mastitis test (CMT) was carried out using the approach outlined by Abdalhamed *et al*. [12] and revealed a positive reaction in 34 milk samples. The subclinical mastitis was collected from 20 cows (80 quarters), which had received no medical treatment for 7–10 days. The CMT-positive milk samples were collected aseptically from each teat and kept in sterile plastic bags before being sent to the laboratory under complete aseptic conditions. A loopful of milk sample was inoculated on 5% sheep blood agar and mannitol salt agar (Oxoid, UK). All plates were incubated for 18–24 h at 37°C before being inspected for bacterial growth. Gram staining, hemolysis patron, catalase response, oxidative-fermentative test, and coagulase tests were all performed on suspected colonies [13].

### Vitek2 compact system method for staphylococci identification

The Vitek2 compact system procedure was carried out as directed by the manufacturer (BioMerieux, France, 2006-Compact ref Manual – Ref-414532) [14].

### Antibiogram profile of CNS isolates

*In vitro*, the disk diffusion method was used to detect CNS isolate’s susceptibility to antibiotics as described by Clinical and Laboratory Standards Institute [15]. The 10 antibiotics employed in this study are C, 30 μg, ciprofloxacin (CIP, 5 μg), clindamycin (DA, 2 μg), P, 10 μg, TE, 30 μg, gentamycin, 120 μg, vancomycin, 30 μg, amoxicillin (AML, 10 μg), OX, 1 μg, and erythromycin (E, 15 μg).

### Detection of the *mecA* gene from isolated CNS by conventional polymerase chain reaction (PCR)

#### DNA extraction

DNA extraction from bacterial cultures was performed using the QIAamp DNA Mini kit (Qiagen, Germany, GmbH) following the instructions.

#### PCR condition and primers sequences used for detection of mecA gene

The PCR reaction was performed using GS-96 gradient thermocycler (Hercuvan, Malaysia) in a final volume of 25 μL. The following primers were used for *mecA* gene detection: Forward, GTA GAAA TGACTGAACGTCCGATAA, and reverse, CCA ATTCCACATTGTTTCGGTCTAA, with an amplicon size of 310 bp [16]. The protocol for the amplification cycles had initial denaturation at 95°C for 4 min, followed by 34 cycles of denaturation at 95°C for 30 s, annealing at 58°C for 40 s, and extension at 72°C for 1 min, with a final extension at 72°C for 10 min.

## Results and Discussion

Subclinical mastitis seems to be more common and results in decreased milk production without any clinical symptoms or milk abnormalities [17]. Recently, non-aureus staphylococci (NAS) have been discovered to be the most common microbe causing subclinical mastitis in dairy cows [18]. The CNS is not only harmful pathogens to human health, but also they can cause a variety of infections in animals, especially in cattle production, resulting in financial losses. In the present study, the prevalence of subclinical mastitis of a total of 80 teat quarters from 20 dairy cows was examined by CMT. Thirty-four positive milk samples (42.5%) were diagnosed with subclinical mastitis. Among the CMT-positive samples, 15 CNS isolates were confirmed with an incidence of 44.12% (15/34 isolates) on the mannitol salt agar medium. In Egypt, El-Seedy *et al*. [19] recorded a total of 95 CNS isolates identified at a proportion of 26.09% from milk samples. Furthermore, El-Jakee *et al*. [20] isolated 76 CNS isolates with an incidence of 16.6% out of 459 subclinical mastitis samples. The frequency of CNS-induced mastitis has been studied all over the world. According to Piepers *et al*. [21], CNS was found to be the cause of more than half of all mastitis in Belgium. Furthermore, Bal *et al*. [22], in a comparable Turkish investigation isolated 100 CNS species from 221 quarter milk samples at a rate of 45.25%. In the present study, *Staphylococcus haemolyticus* was the most predominantly isolated species with an incidence of 33.3% (5/15 isolates), followed by 26.7% (4/15 isolates) for *Staphylococcus sciuri* and *Staphylococcus vitamins*, and 13.3% (2/15 isolates) for *Staphylococcus vitulinus* (2/15), respectively [22]. Schmidt *et al*. [23] stated that *Staphylococcus chromogenes*, *Staphylococcus xylosus*, *Staphylococcus simulans*, *Staphylococcus epidermidis*, *S. haemolyticus*, and *S. sciuri* are the most frequently isolated bovine CNS. In addition, *S. chromogenes*, *S. sciuri*, and *S. haemolyticus* were recorded as the three major CNS species isolated from Polish cows’ milk [24]. Furthermore, in a similar study, *S. xylosus*, *S. chromogenes*, *S. haemolyticus*, *S. simulans*, and *S. sciuri* were the most abundant species in subclinical mastitis milk samples [25]. Jenkins *et al*. [26] found that *S. chromogenes* was the most prevalent CNS species in many dairy herds in the United States, followed by *S. haemolyticus*, *S. simulans*, *S. epidermidis*, *Staphylococcus hominis*, *Staphylococcus auricularis*, *S. sciuri*, *Staphylococcus devriesei*, *Staphylococcus capitis*, *Staphylococcus cohnii*, and *Staphylococcus warneri*. Furthermore, Andrade-Becerra *et al*. [27] mentioned that *S. epidermidis*, *S. chromogenes*, *S. sciuri*, *S. simulans*, *S. haemolyticus*, and *S. capitis* were the most frequent species in bovine mastitis cases. Moreover, all of these studies found that the species that can infect the mammary gland were consistent regardless of region and that this similarity could be related to similar management strategies among the herds studied. In addition, management control can be utilized to fight infections in different countries to achieve similar results. Pathogenicity, persistence, and dissemination of CNS in cows are all linked to a variety of variables. For example, some species, such as *S. chromogenes* and *S. haemolyticus*, may have colonization advantages in the mammary gland because of hemolytic and proteolytic activity, better tolerance to post-milking teat disinfection, and the capacity to elude the immune system [28]. Our findings are consistent with the reports from the other regions of the world, indicating that these pathogens must be checked regularly, especially in farms where major pathogens are not a serious concern.

The European Union plans to limit livestock antimicrobial sales by 50% by 2030 under the “Farm to Fork” strategy, due to growing bacterial pathogen resistance and the prevalence of MR staphylococci (MRS) in veterinary medicine [29]. One of the most important characteristics of pathogenicity is staphylococci’s capacity to resist antimicrobials, which permits these infections to attach to and colonize the mammary gland epithelium with poor antimicrobial therapy [30].

Table-1 shows the antibiotic sensitivity test. The present study reported that the CNS tested isolates showed the highest resistance rate of 40% against P and OX (6/15 isolates), followed by 33.3% for DA (5/15 isolates), 13% for C, AML, and E (2/15 isolates), and 5% for CIP (1/15 isolates), respectively. Our results revealed that the isolates were resistant to one or more antimicrobial agents.

**Table-1:** Antibiogram of 15 coagulase-negative staphylococci tested isolates.

Antibiotic and concentration	Antibiotic profile			

	Resistant		Sensitive	
	
	No.	%	No.	%
Chloramphenicol (30 μg)	2	13.3	13	86.7
Ciprofloxacin (5 μg)	1	6.6	14	93.3
Clindamycin (2 μg)	5	33.3	10	66.7
Penicillin (10 μg)	6	40	9	60
Tetracycline (30 μg)	3	20	12	80
Gentamycin (120 μg)	1	6.6	14	93.3
Vancomycin (30 μg)	2	13.3	13	86.7
Amoxicillin (10 μg)	2	13.3	13	86.7
Oxacillin (1 μg)	6	40	9	60
Erythromycin (15 μg)	2	13.3	13	86.7

**Figure-1:**
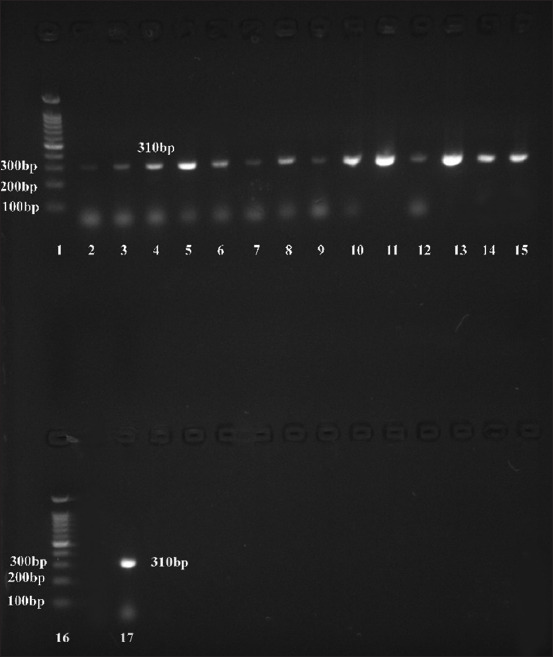
Agarose gel electrophoresis of polymerase chain reaction product amplified from methicillin-resistant *Staphylococcus aureus* *mecA* gene (310 bp). Lanes 1 and 16, 100 bp DNA ladder; lanes, 2–15 and 17, positive samples.

Furthermore, multiple antimicrobial resistance was observed in five isolates. According to Condas *et al*. [31], this observation is significant as MRS strains associated with resistance to multiple antibiotic classes pose a considerable threat to public health. The MRS, particularly those resistant to β-lactam antibiotics, have been detected in raw milk and dairy products, including cheese, according to recent studies [32]. According to the World Health Organization, MRS strains’ opportunistic capacity to cause mastitis poses a health risk to the public. They have the potential to spread zoonotic diseases and provide antibiotic resistance genes to humans who interact with dairy cows, and vice versa [33]. Although *S. aureus* (MRSA) is the most usually reported MRS, NAS have also been identified as MRS isolates in several studies [34]. The presence of MR-CNS strains is described as a significant source of worry because of the potential for their spread through the dairy food chain. The MR is predominantly imparted in staphylococci through the replication of the *mecA* gene, which complicates the treatment of MR-CNS infections and constitutes a public health risk [35]. Figure-1 shows all the CNS isolates harbored the *mecA* gene with an incidence of 100%.

Nayel *et al*. [10] reported that the *mecA* gene was detected in 73.33% of CNS isolates from mastitic cows, and interestingly, 26.67% of *mecA* negative isolates were OX resistant phenotypically which is in agreement with our findings. Furthermore, Walid *et al*. [36] reported that *mecA* was the most identified gene (73.3%) from CNS isolates that exhibited multidrug resistance (MDR) in bovine mastitis in dairy herds [36]. The present study shows a higher incidence of *mecA* than empirical research, which effectively detected *mecA* with prevalence rates of 9.7% [37]. Klimiene *et al*. [4] found that 23.8% of CNS isolates from bovine mastitis carried the *mec*A gene. Furthermore, it is not surprising that only six CNS isolates out of 15 phenotypically demonstrated OX resistance, according to our research. The most likely explanation is the phenotypic expression of resistance varies depending on growth conditions (such as osmolarity or medium temperature), making susceptibility testing of MRS by normal microbiological methods challenging. This explanation was confirmed by Bogado *et al*. [38] who investigated the disparities between molecular and phenotypic classifications of methicillin resistance. Furthermore, Moon *et al*. [39] exhibited that isolates lacking the *mecA* gene were phenotypically MR. However, the presence of MR *S. epidermidis* strains in bovine milk samples is a matter of concern as they may act as a reservoir of genetic elements carrying antimicrobial resistance. SCCmec elements can be transferred to other staphylococci species, including *S. aureus*, due to their mobile nature [40]. Therefore, in the absence of the *mecA* gene, overproduction of β-lactamase, production of a new methicillinase, or alterations in PBPs could all explain the phenotype of resistance to OX.

## Conclusion

The present study on the antibiograms and molecular features of *Staphylococcus* spp. revealed a widespread and increasing trend of methicillin resistance and multiple resistance to other antibiotics. In addition, this implies that to combat and control the spread of these pathogens effectively, the “One Health” practice should be facilitated not only at the dairy farm level but also at the national or even international levels through the collaboration of different sectors (dairy farmers, veterinarians, medical and public health personnel, and scientists). Furthermore, it is recommended to implement control measures such as hygiene regimen, regular screening for MDRCNS screening, application of antibiotic susceptibility testing before treatment, or random selection of antibiotics in field cases to avoid the emergence of resistance phenomenon.

## Authors’ Contributions

ESI and SMD: Conceived and designed the study, performed the study, analyzed the data, and drafted and revised the manuscript. ASM, AMA, and DDK: Carried out sample collections and laboratory analysis, data collection, and analysis. All authors have read and approved the final manuscript.
